# Higher Neutrophil-To-Lymphocyte Ratio Was Associated with Increased Risk of Chronic Kidney Disease in Overweight/Obese but Not Normal-Weight Individuals

**DOI:** 10.3390/ijerph19138077

**Published:** 2022-06-30

**Authors:** Chia-Ho Lin, Yu-Hsuan Li, Ya-Yu Wang, Wen-Dau Chang

**Affiliations:** 1 Department of Medical Education, Taichung Veterans General Hospital, Taichung 40705, Taiwan; stevenlin110101@gmail.com; 2 Division of Endocrinology and Metabolism, Department of Internal Medicine, Taichung Veterans General Hospital, Taichung 40705, Taiwan; brightlight@vghtc.gov.tw; 3 Department of Family Medicine, Taichung Veterans General Hospital, 1650 Taiwan Boulevard Section 4, Taichung 40705, Taiwan; yyywang2001@gmail.com; 4 Department of Veterinary Medicine, College of Veterinary Medicine, National Chung Hsing University, Taichung 402202, Taiwan; 5 School of Medicine, National Yang Ming Chiao Tung University, Taipei 112304, Taiwan; 6 Department of Post-Baccalaureate Medicine, College of Medicine, National Chung Hsing University, Taichung 402202, Taiwan

**Keywords:** chronic kidney disease, inflammation, neutrophil to lymphocyte ratio, overweight, obesity

## Abstract

*Background*: Inflammation has been proposed to play potential roles in the development and progression of chronic kidney disease (CKD). We evaluated the relationship of neutrophil-to-lymphocyte ratio (NLR), a systemic inflammation marker, with CKD in normal-weight and overweight/obese adults. *Methods*: This cross-sectional study included 2846 apparently healthy adults who underwent a health examination between August 2000 and April 2002. Normal-weight was defined as a body mass index (BMI, kg/m^2^) of 18.5–24, while overweight/obesity was defined as a BMI of ≥24. CKD was defined as an estimated glomerular filtration rate (eGFR) of <60 mL/min/1.73 m^2^. Logistic and linear regression analysis was performed to explore the NLR–CKD relationship. *Results*: Of the 2846 participants (1777 men and 1069 women), there were 348 CKD individuals (12.3%), with 262 (14.7%) men and 86 (8%) women. A total of 1011 men (56.9%) and 408 women (38.2%) were overweight or obese. Compared with the normal-weight participants, CKD prevalence was higher in the overweight/obese women (6.1% vs. 11.3%, *p* = 0.002), but not in the overweight/obese men (14.5% vs. 14.9%, *p* = 0.793). CKD percentages in the NLR quartile groups were 9.4%, 11.5%, 15.4%, and 22.7% in men (*p* < 0.0001) and 6.4%, 7.1%, 10.5%, and 8.2% in women (*p* = 0.2291). After adjustment for confounders, each increment of one unit of NLR was associated with a higher CKD risk in the overweight/obese men (adjusted odds ratio (OR) = 1.37, 95% confidence interval (CI) = 1.03–1.82, *p* = 0.03) and women (adjusted OR = 1.77, 95% CI = 1.08–2.90, *p* = 0.023), whereas NLR was not associated with CKD in normal-weight men or women. Further, in the overweight/obese participants with an eGFR of 50–70 mL/min/1.73 m^2^, univariable linear regression analysis revealed a significant negative correlation between NLR and eGFR for men (*p* = 0.004) and women (*p* = 0.009). *Conclusions*: It was found that higher NLR was associated with an increased CKD risk in overweight/obese but not in normal-weight men and women in an adult health examination dataset. Our study suggests a role of NLR for CKD prediction in overweight/obese individuals.

## 1. Introduction

Chronic kidney disease (CKD) is a significant global health issue, which can lead to several morbidities and eventually mortality, placing a heavy burden on the healthcare system [1]. Among several known traditional risk factors of CKD (e.g., diabetes, hypertension, and dyslipidemia), it is known that inflammation, regardless of CKD etiology, is also likely to be a key cause and consequence of almost all kidney diseases [2]. Serum concentrations of inflammatory markers are elevated in patients with CKD compared with those with normal kidney function, and they are associated with worsening kidney function in the general population [3,4]. Furthermore, many studies have demonstrated that body mass index (BMI, kg/m^2^) is a potential predictor of proteinuria, glomerular filtration rate (eGFR) decline, and development of end-stage renal disease [5,6,7]. The increased risk for CKD among obese individuals, in addition to the underlying cardiometabolic abnormalities, can be due to excess adiposity-associated chronic inflammatory process, which contributes to early pathogenic mechanisms of kidney injury [7]. However, some studies reported negative roles of obesity for CKD on an inflammatory basis [8,9,10]. It is proposed that inflammation profiles may be modified by the associated metabolic factors according to body weight status [9,10]. Accordingly, a further examination of the inflammatory role for CKD in normal-weight or overweight/obese individuals is necessary.

The neutrophil-to-lymphocyte ratio (NLR), a surrogate marker for systemic inflammation, has recently gained increasing public interest. NLR is associated with several comorbidities, including insulin resistance and cardiovascular disease (CVD) [11,12]. It is also significantly associated with the prevalence and incidence of type 2 diabetes, and it has been suggested as a potential T2D biomarker [13]. Furthermore, NLR increases with CKD progression [14,15]. However, whether the association between NLR and CKD can be altered on the basis of BMI has been less studied. Taiwan faces a high prevalence of CKD [16,17], which can increase end-stage renal disease, placing an economic burden on the healthcare system [1]. Moreover, in Taiwan, the prevalence of overweight and obesity was reported to be 44.1% among all adults (50.8% in men and 36.9% in women), according to previously published data with overweight defined as a BMI of 24–26.9 kg/mm^2^, and obesity defined as a BMI ≥ 27 kg/m^2^ [18,19]. Considering the increasing epidemic of CKD and obesity in Taiwan, and the potential roles of inflammation and obesity in the pathogenesis of CKD, the present study investigated the relationships among NLR, body mass index, and CKD. It was aimed at examining the predictive value of NLR for CKD in a relatively healthy adult population categorized according to BMI as normal-weight and overweight/obese.

## 2. Materials and Methods

### 2.1. Study Design and Participants

This was a hospital-based cross-sectional study, designed according to ‘Strengthening the Reporting of Observational Studies in Epidemiology’ (STROBE) guidelines [20]. Participants were enrolled from a population that underwent a self-paid packaged physical examination at a medical center in central Taiwan between August 2000 and April 2002, and the data were registered in the healthcare database of Taichung Veterans General Hospital. Because all data were retrospectively analyzed anonymously, verbal or written consent was not required from the participants according to the regulations of the hospital’s ethics committee. This study was approved by the Institutional Review Board of Taichung Veterans General Hospital (protocol no: CE17066B).

Among 4832 adult participants (age ≥ 20 years) who attended the physical examination, those with an incomplete questionnaire (*n* = 1423) or missing data for blood pressure (BP) measurement (*n* = 205), serum creatinine (SCr, *n* = 1), serum uric acid (*n* = 4), total WBC count (*n* = 1), and WBC differential count (*n* = 6) were excluded. Participants with a total WBC count of ≥10,000 cells/μL (*n* = 125) and <3000 cells/μL (*n* = 6) due to possible active infection or hematological disorders, previous analgesic use (*n* = 77), or a history of cancer (*n* = 30) were excluded. To preclude potential undernourishment and associated morbidity, underweight participants (BMI < 18.5; *n* = 108, 38 men and 70 women) were also excluded. Finally, 2846 participants (1777 men and 1069 women) were included in the analysis.

### 2.2. Data Acquisition

On the day of the health examination, clinical data, including age, sex, chronic diseases (hypertension, diabetes mellitus (DM), myocardial infarction, and stroke), medication use, and lifestyle habits, such as smoking and alcohol consumption, were all collected using a structured questionnaire. For smoking status, the participants were categorized as nonsmokers (never and former smokers) and current smokers. For alcohol consumption status, the participants were classified as nondrinkers (less than one intake/week) and habitual drinkers (at least one intake/week). BP was measured using standard mercury sphygmomanometers when the participants were in a sitting position after 5 min of rest. A diagnosis of hypertension was made if participants had a previous history of hypertension or taking antihypertensive medications, or if their systolic BP was ≥140 mmHg or diastolic BP was ≥90 mmHg. DM was diagnosed if participants had a previous history or taking antidiabetic medications, or if their fasting plasma glucose concentration was ≥7 mmol/L (126 mg/dL). Hypercholesterolemia was defined as a total cholesterol concentration of ≥5.18 mmol/L (200 mg/dL), while hypertriglyceridemia was defined as a triglyceride concentration of ≥1.70 mmol/L (150 mg/dL) or a low high-density lipoprotein-cholesterol (HDL-C) < 1.04 mmol/L (40 mg/dL) for men and <1.29 mmol/L (50 mg/dL) for women. Dyslipidemia was defined as any of the aforementioned lipid abnormalities or the use of lipid-lowering drugs [21].

Venous blood samples of the participants were collected in the morning after an overnight fast (≥8 h) to measure glucose, total bilirubin, aspartate aminotransferase (AST), alanine aminotransferase (ALT), total cholesterol, triglyceride, HDL-C, and uric acid concentrations using a chemical analyzer (Hitachi 7600, Tokyo, Japan) at the central laboratory of the hospital.

### 2.3. Anthropometric Measurement and Definition of Overweight and Obesity

All participants were weighed in light clothing with no shoes, and their heights were also measured. BMI was calculated as weight (in kilograms) divided by height (in meters) squared. Individuals with BMI ≥ 24, BMI between 18.5 and 24, and BMI < 18.5 were defined as overweight/obese, normal-weight, and underweight, respectively, according to the guidelines of Ministry of Health and Welfare, Taiwan [19].

### 2.4. Measurement of Inflammation from Blood Cell Counts

Total WBC count, WBC differential count, and hemoglobin level were computed using an autoanalyzer (Sysmex SE-9000, Kobe, Japan). The NLR was calculated as neutrophil counts (cells/μL) divided by lymphocyte counts (cells/μL).

### 2.5. Renal Function Measurement and CKD Definition

Serum creatinine (SCr) was measured using the Jaffe method (Hitachi 7600) in the hospital laboratories. The estimated glomerular filtration rate (eGFR) was calculated using the CKD-EPI equation: eGFR (mL/min/1.73 m^2^) = 141 × min(SCr/κ, 1)^α^ × max(SCr/κ, 1)^−1.209^ × 0.993^age^ × 1.018 (if females), where κ is 0.7 for females and 0.9 for males, α is −0.329 for females and −0.411 for males, and min and max indicate the minimum and maximum of SCr/κ or 1, respectively [22]. Individuals with eGFR < 60 mL/min/1.73 m^2^ were defined as having CKD [23].

### 2.6. Statistical Analysis

Continuous and categorical variables are presented as the mean ± standard deviation (SD) and number (percentage), respectively. Between-group comparisons were performed using the chi-square test or Fisher’s exact test for categorical variables and the unpaired *t*-test, ANOVA test, Mann–Whitney U test, or Kruskal–Wallis H test for continuous variables. To assess trends across ordered groups, nonparametric tests for trends and a score test for the trend of odds were used. To investigate the effects of body weight on CKD, the participants were divided into normal-weight (18 ≤ BMI < 24) and overweight/obesity (BMI ≥ 24) groups. The eGFR values and CKD prevalence between the two BMI groups were determined in male and female participants. To evaluate the relationship between NLR and CKD, the participants were divided into NLR quartiles, and the eGFR and CKD prevalence were examined. Furthermore, the prevalence in the four NLR groups was compared between CKD and non-CKD participants in normal-weight and overweight/obese individuals. To evaluate the independent association between NLR and CKD, multivariable logistic regression analysis was performed after adjustment for conventional CKD risk factors (sex, age, BMI, smoking and alcohol consumption, systolic BP, and fasting plasma glucose, total cholesterol, triglyceride, HDL-C, ALT, uric acid, total bilirubin, and hemoglobin levels). The correlation between NLR and eGFR was examined by multivariable linear regression analysis after adjustment of the potential confounders.

All statistical analyses were performed using the STATA 10 software (StataCorp. 2007. Stata Statistical Software: Release 10. StataCorp LP: College Station, TX, USA), and a two-tailed *p*-value < 0.05 was considered statistically significant.

## 3. Results

### 3.1. CKD in Different Weight Groups

The characteristics of male and female participants are shown in Table 1A,B, respectively. Of the 2846 participants (1777 men and 1069 women), there were 348 CKD individuals (12.3%), with 262 (14.7%) men and 86 (8%) women. A total of 1011 men (56.9%) and 408 women (38.2%) were overweight or obese. Compared with the normal-weight participants, CKD prevalence was higher in the overweight/obese women (40/661 vs. 46/408 or 6.1% vs. 11.3%, *p* = 0.002), but not in the overweight/obese men (111/766 vs. 151/1011 or 14.5% vs. 14.9%, *p* = 0.793).

Compared with the normal-weight men, the overweight/obese men were younger and had higher BP, total cholesterol, triglyceride, AST, ALT, hemoglobin, uric acid levels, and total WBC count and lymphocyte count, but lower HDL-C and NLR levels (Table 1A). In addition, the overweight/obese men had a higher prevalence of hypertension, dyslipidemia, and habitual alcohol consumption. Compared with the normal-weight women, the overweight/obese women were older and had higher BP, fasting glucose, total cholesterol, triglyceride, uric acid, ALT, hemoglobin levels, and total WBC count, neutrophil count, and lymphocyte count, but lower HDL-C, eGFR, and total bilirubin levels (Table 1B). Moreover, the overweight/obese women had a higher prevalence of hypertension, DM, and dyslipidemia.

Compared with the normal-weight participants, CKD prevalence was higher in the overweight/obese women (40/661 vs. 46/408 or 6.1% vs. 11.3%, *p* = 0.002), but not in the overweight/obese men (111/766 vs. 151/1011 or 14.5% vs. 14.9%, *p* = 0.793).

### 3.2. CKD in NLR Quartile Subgroups

The characteristics of male and female participants according to blood NLR quartiles are shown in Table 2A,B, respectively. The prevalence of hypertension and CVD in men increased with increasing blood NLR levels. In men, age, systolic and diastolic BP, prevalence of hypertension and CVD, and blood creatinine significantly increased with higher NLR levels (all *p* < 0.05), whereas eGFR, BMI, prevalence of habitual drinker, and AST, ALT, hemoglobin levels significantly decreased with higher NLR levels (all *p* < 0.05) (Table 2A). Furthermore, CKD percentages in the NLR quartile groups were 9.4%, 11.5%, 15.4%, and 22.7% for men (trend *p* < 0.0001). In women, only the cholesterol level and the prevalence of current smoker decreased significantly with increasing NLR levels (Table 2B). CKD percentages in the NLR quartile groups were 6.4%, 7.1%, 10.5%, and 8.2% for women (trend *p* = 0.2291). According to different body weight status, the percentage of higher NLR was significantly increased in normal-weight or overweight/obese men with CKD in comparison with that in non-CKD men, and there was a nonsignificant higher NLR percentage in overweight/obese women (Table 3).

### 3.3. Relationship between NLR and CKD, Stratified by Body Weight Status

In all men and women with BMI ≥ 24 (*n* = 1419), multivariable logistic regression analysis (after adjustment for the conventional CKD risk factors) showed that each increment of one unit of NLR was associated with an increased CKD risk (adjusted odds ratio (OR) = 1.45, 95% confidence interval (CI) = 1.13–1.84, *p* = 0.003), and the CKD risk did not differ between men and women (adjusted OR = 1.17, 95% CI = 0.67–2.02, *p* = 0.580 for men vs. women) (Table 4A). In participants with a BMI of 18–24 (*n* = 1427), no association was noted between NLR and CKD (adjusted OR = 0.96, 95% CI = 0.77–1.20, *p* = 0.744), although men had a higher CKD risk than women (adjusted OR = 2.62, 95% CI = 1.43–4.82, *p* = 0.002) (Table 4A). We further assessed the NLR–CKD relationship, stratified by the BMI status separately in men and women (Table 4B and Table 4C, respectively). Multivariable logistic regression analysis revealed that the positive association between NLR and CKD remained significant in both men and women with BMI ≥ 24 after adjustment for the conventional CKD risk factors. Every additional unit of NLR was associated with a higher CKD risk in the overweight/obese men (adjusted OR = 1.37, 95% CI = 1.03–1.82, *p* = 0.030) and women (adjusted OR = 1.77, 95% CI = 1.08–2.90, *p* = 0.023), whereas no association was observed between NLR and CKD among the normal-weight men (adjusted OR = 0.97, 95% CI = 0.74–1.29, *p* = 0.846) or women (adjusted OR = 0.87, 95% CI = 0.53–1.45, *p* = 0.601).

**Figure 1:**
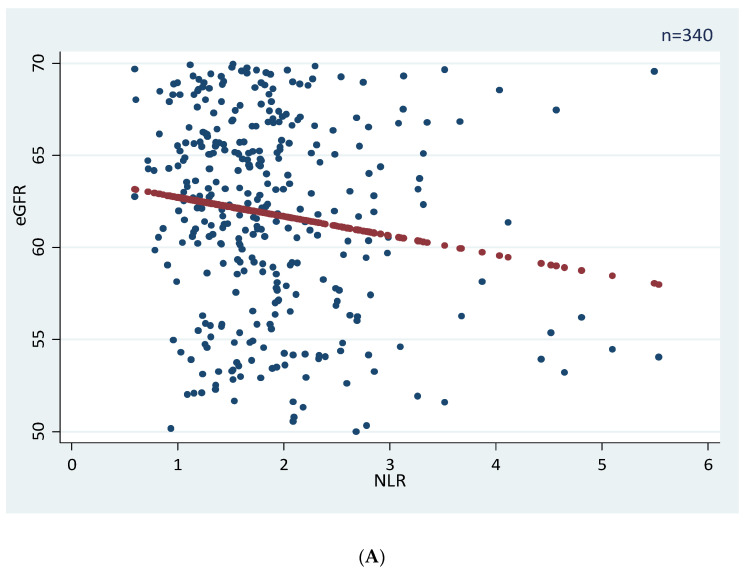

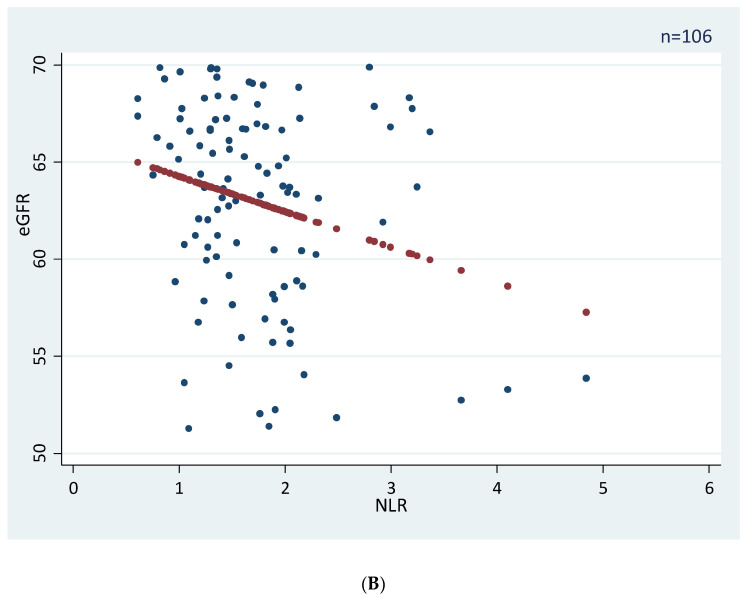
(**A**) Linear regression analysis of eGFR between 50 and 70 mL/min/1.73 m^2^ against NLR in overweight/obese men with eGFR as the dependent variable. The mathematical equation was eGFR = 63.8 − 1.04 × NLR with a slope of −1.04 (95% CI: −1.75 to −0.34; *p* = 0.004), indicating a mean eGFR decrease of 1.04 mL/min/1.73 m^2^ per unit NLR increment. (**B**) Linear regression analysis of eGFR between 50 and 70 mL/min/1.73 m^2^ against NLR in overweight/obese women with eGFR as the dependent variable. The mathematical equation was eGFR = 66.1 − 1.82 × NLR with a slope of −1.82 (95% CI = −3.17 to −0.47; *p* = 0.009), indicating a mean eGFR decrease of 1.04 mL/min/1.73 m^2^ per unit NLR increment.

In overweight/obese men and women, multivariable linear regression analysis revealed that eGFR correlated negatively with age and blood uric acid levels, but did not correlate with NLR (Table 5A for men and Table 5B for women). However, for eGFR between 50 and 70 mL/min/1.73 m^2^, there was a significant negative correlation between NLR and eGFR in overweight/obese men (coefficient = −1.04, 95% CI = −1.75 to −0.34, *p* = 0.004) and overweight/obese women (coefficient = −1.82, 95% CI = −3.17 to −0.47, *p* = 0.009) using the univariable linear regression analysis (Figure 1A for men and Figure 1B for women).

## 4. Discussion

In the present study, after adjustment for the conventional CKD risk factors, we found that higher NLR was independently associated with a higher CKD risk in both the overweight/obese (BMI ≥ 24) men and women. By contrast, no such positive association was observed in the normal-weight (BMI = 18.5–24) men or women.

Studies have reported that, in obesity, nutrition overload can lead to cytokine release in adipose tissue, subsequently triggering an immune response [24]. It is proposed that neutrophils represent the innate immune system and lymphocytes represent the adaptive immune system; thus, NLR can represent the integration of these two immune pathways [12]. In line with previous studies, we observed that an increase in BMI was associated with increased WBC, lymphocyte, and neutrophil counts in both genders [25,26]. An explanation for NLR in overweight/obese men and women not being higher was possibly due to the concomitant increase in lymphocyte and neutrophil counts. Clinically, NLR has been reported to be associated with various comorbidities, such as hypertension, insulin resistance, and type 2 diabetes [12,13,14,15]. Furthermore, some studies have shown that NLR can effectively predict diabetic vascular complications, nonalcoholic fatty liver disease [27,28], and clinical progression of CKD [14,15,29,30]. In this study, we additionally found that NLR was associated with CKD, particularly in an overweight/obesity population. Generally, it has been proposed that inflammation may be a potential pathogenic factor in the CKD [2]. However, it should be noted the effects of inflammation on CKD may be modified by the associated metabolic factors according to body weight status [8,9,10]. In our study, the association between NLR and CKD remained significant after adjustment of the other potential confounders in participants with overweight and obesity. This suggested that NLR may be used as a potential predictor of CKD in overweight/obese individuals.

The reasons for NLR not being associated with CKD in normal-weight people are unclear. A similar observation indicated that CRP is not predictive of CKD in people with a normal BMI [31]. In many studies, inflammatory biomarker levels were mostly determined on the basis of a single measurement and may not represent the average levels over time because the concentration of these biomarkers could be affected by many factors, such as metabolic factors (e.g., obesity and diabetes), environmental factors, and underlying infections. Moreover, body central fat distribution is considered a more critical risk factor for CKD than BMI, and reliance on BMI alone might underestimate the associated risk [31]. A recent paper reported that additional use of body fat composition data enhanced the capacity of WBC level changes to predict the risk of chronic diseases, such as CKD [32]. In addition, another study showed that men and women having both high BMI and high waist had the highest risk of elevated CRP levels compared to their counterparts [33]. Since different obesity indices may modify inflammation, further studies identifying various body composition and inflammatory states are warranted to elucidate the role of inflammation in CKD.

In the present study, we investigated men and women separately because prior evidence suggested sexual dimorphism in CKD development [34,35]. In normal-weight participants, CKD risk was less in women than in men, indicating the gender difference in CKD progression. However, in the overweight and obese subjects, the risk of CKD did not differ between men and women. Published studies have demonstrated higher obesity-related inflammation in women than in men because women usually have greater body fat composition, whereas men have more lean muscle mass [36,37]. It was proposed that increased inflammation in overweight/obese women may decrease the protective effect of estrogen on glomerular cells, making them more susceptible to obesity-induced inflammation and consequent kidney injury [38]. In this study, the odds ratio of CKD by NLR was higher in overweight and obese women than in men, seemingly supporting this hypothesis. Overall, using inflammatory biomarkers (e.g., NLR) as a risk factor for CKD may be complex because it can be affected by gender, age, or obesity status [39], and prospective studies are required to more clearly define how inflammation interferes with CKD development.

Our study had several limitations. Firstly, this study had a cross-sectional design; thus, establishing a causal relationship between NLR and CKD was not possible. Systemic inflammation may have led to kidney dysfunction or decreased kidney filtration, resulting in elevated inflammatory biomarkers, or their interactions contributed to the observed study findings. Secondly, we did not assess other inflammatory markers such as CRP. In addition, only one NLR measurement was included in the analysis. Although we excluded participants with total WBC count of ≥10,000 cells/μL and <3000 cells/μL, this did not exclude the possibility of an acute and brief episode of infection or bone marrow disorder leading to bias in the observed correlation. Thirdly, this study used only the CKD-EPI equation to define renal function rather than a more direct measurement of GFR, such as insulin clearance. However, the latter method, albeit a more precise measure of renal function, is laborious in large epidemiological studies and in clinical practice. Fourthly, our study did not include urine protein data, which might have influenced the accurate estimation of CKD proposed by the NKF-KDOQI [40]. Lastly, regarding socioeconomic factors, race, and culture, our study participants may not be representative of the general population because they were volunteers for a self-paid health examination at a single hospital.

## 5. Conclusions

In overweight/obese (BMI ≥ 24) men and women from an adult health examination dataset, we found that higher NLR was associated with a higher CKD risk, regardless of conventional CKD risk factors. By contrast, among the normal-weight men and women (BMI = 18.5–24), NLR was not associated with CKD. Our study suggests a role of NLR for CKD prediction in overweight/obese individuals.

## Figures and Tables

**Table 1:** Characteristics of Men (**A**) and Women (**B**).

(A) Men				
	All (*n* = 1777)	Normal Weight 18.5 ≤ BMI < 24 (*n* = 766)	Overweight/Obesity BMI ≥ 24 (*n* = 1011)	*p*-Value
BMI (kg/m^2^)	24.7 ± 2.94	22.1 ± 1.40	26.6 ± 2.21	<0.0001
Age (years)	51.2 ± 11.8	51.8 ± 12.4	50.7 ± 11.2	0.0466
Systolic BP (mmHg)	122.6 ± 17.3	120.0 ± 16.8	124.6 ± 17.4	<0.0001
Diastolic BP (mmHg)	76.6 ± 11.3	74.1 ± 10.9	78.5 ± 11.2	<0.0001
Fasting glucose (mg/dL)	101.8 ± 30.5	100.4 ± 33.2	102.9 ± 28.2	0.1010
Total cholesterol (mg/dL)	194.7 ± 35.5	191.4 ± 35.6	197.2 ± 35.2	0.0006
Triglyceride (mg/dL)	147.0 ± 131.8	119.4 ± 84.3	167.9 ± 155.4	<0.0001
HDL-C(mg/dL)	51.6 ± 12.6	54.8 ± 13.6	49.1 ± 11.2	<0.0001
AST (U/L)	26.7 ± 13.9	25.3 ± 13.7	27.7 ± 14.0	0.0003
ALT (U/L)	32.7 ± 25.5	27.7 ± 23.4	36.5 ± 26.4	<0.0001
Creatinine (mg/dL)	1.17 ± 0.23	1.16 ± 0.24	1.17 ± 0.22	0.2201
eGFR (mL/min/1.73 m^2^)	74.5 ± 14.6	74.8 ± 14.7	74.2 ± 14.5	0.3857
eGFR <60 mL/min/1.73 m^2^	262 (14.7)	111 (14.5)	151 (14.9)	0.793
Uric acid (mg/dL)	7.0 ± 1.52	6.7 ± 1.51	7.2 ± 1.49	<0.0001
Total bilirubin (mg/dL)	0.87 ± 0.39	0.88 ± 0.39	0.86 ± 0.39	0.5623
Hemoglobin (g/dL)	15.0 ± 1.24	14.8 ± 1.27	15.2 ± 1.19	<0.0001
Total WBC (cells/μL)	6400 ± 1362	6302 ± 1404	6473 ± 1324	0.0087
Neutrophil (cells/μL)	3581 ± 1040	3582 ± 1090	3581 ± 1001	0.9935
Lymphocyte (cells/μL)	2162 ± 594	2079 ± 596	2225 ± 585	<0.0001
Neutrophil/lymphocyte ratio	1.78 ± 0.74	1.85 ± 0.80	1.72 ± 0.69	0.0002
Previous CVD	46 (2.6)	19 (2.5)	27 (2.7)	0.803
Hypertension	560 (31.5)	194 (25.3)	366 (36.2)	<0.001
Diabetes mellitus	187 (10.5)	78 (10.2)	109 (10.8)	0.684
Dyslipidemia	1076 (60.6)	415 (54.2)	661 (65.4)	<0.001
Current smoker	584 (32.9)	248 (32.4)	336 (33.2)	0.703
Habitual drinker	552 (31.1)	212 (27.7)	340 (33.6)	0.007
**(B) Women**				
	**All** **(*n* = 1069)**	**Normal Weight** **18.5 ≤ BMI < 24** **(*n* = 661)**	**Overweight/Obesity** **BMI ≥ 24** **(*n* = 408)**	***p*** **-Value**
BMI (kg/m^2^)	23.5 ± 3.13	21.6 ± 1.41	26.7 ± 2.45	<0.0001
Age (years)	48.7 ± 11.3	46.1 ± 11.3	52.9 ± 9.8	<0.0001
Systolic BP (mmHg)	118.1 ± 19.4	113.5 ± 17.0	125.7 ± 20.7	<0.0001
Diastolic BP (mmHg)	73.0 ± 11.8	70.5 ± 10.8	77.1 ± 12.2	<0.0001
Fasting glucose (mg/dL)	95.4 ± 21.9	91.6 ± 13.0	101.5 ± 30.5	<0.0001
Total cholesterol (mg/dL)	192.8 ± 36.4	188.4 ± 35.1	200.1 ± 37.5	<0.0001
Triglyceride (mg/dL)	108.9 ± 174.7	87.9 ± 53.4	143.0 ± 271.2	<0.0001
HDL-C (mg/dL)	63.3 ± 15.5	66.5 ± 15.7	58.0 ± 13.7	<0.0001
AST (U/L)	24.1 ± 26.0	23.7 ± 29.8	24.9 ± 18.5	0.3812
ALT (U/L)	24.4 ± 36.5	22.3 ± 39.6	27.7 ± 30.5	0.0121
Creatinine (mg/dL)	0.89 ± 0.49	0.89 ± 0.43	0.90 ± 0.59	0.7510
eGFR (mL/min/1.73 m^2^)	81.1 ± 16.6	82.8 ± 16.4	78.3 ± 16.4	<0.0001
eGFR <60 mL/min/1.73 m^2^	86 (8.0)	40 (6.1)	46 (11.3)	0.002
Uric acid (mg/dL)	5.5 ± 1.29	5.2 ± 1.17	5.9 ± 1.34	<0.0001
Total bilirubin (mg/dL)	0.67 ± 0.29	0.69 ± 0.31	0.64 ± 0.26	0.0015
Hemoglobin (g/dL)	12.9 ± 1.29	12.8 ± 1.31	13.1 ± 1.23	<0.0001
Total WBC (cells/μL)	6133 ± 1447	5989 ± 1426	6367 ± 1451	<0.0001
Neutrophil (cells/μL)	3468 ± 1098	3407 ± 1119	3566 ± 1059	0.0214
Lymphocyte (cells/μL)	2114 ± 612	2046 ± 580	2224 ± 646	<0.0001
Neutrophil/lymphocyte ratio	1.76 ± 0.84	1.79 ± 0.93	1.71 ± 0.67	0.1215
Previous CVD	16 (1.5)	7 (1.1)	9 (2.2)	0.193
Hypertension	257 (24.0)	88 (13.3)	169 (41.4)	<0.001
Diabetes mellitus	63 (5.9)	19 (2.9)	44 (10.8)	<0.001
Dyslipidemia	581 (54.4)	300 (45.4)	281 (68.9)	<0.001
Current smoker	42 (3.9)	32 (4.8)	10 (2.5)	0.051
Habitual drinker	51 (4.8)	32 (4.8)	19 (4.7)	0.891

Values are expressed as the mean ± standard deviation or number (percentage). BMI, body mass index; BP, blood pressure; HDL-C, high-density lipoprotein cholesterol. AST, aspartate aminotransferase; ALT, alanine aminotransferase; CVD, cardiovascular disease (including stroke and myocardial infarction).

**Table 2:** Characteristics Represented across Quartiles of NLR in Men (**A**) and Women (**B**).

(A) Men					
	NLR Q1 0.40–1.28 (*n* = 445)	NLR Q2 1.29–1.62 (*n* = 444)	NLR Q3 1.63–2.05 (*n* = 443)	NLR Q4 2.06–8.70 (*n* = 445)	*p* for Trend
BMI (kg/m^2^)	24.7 ± 2.81	24.8 ± 2.81	24.8 ± 3.06	24.4 ± 3.06	0.006
Age (years)	48.2 ± 10.6	49.9 ± 11.6	51.1 ± 11.6	55.4 ± 12.1	<0.001
Systolic BP (mmHg)	119.9 ± 15.4	120.7 ± 15.8	124.2 ± 18.7	125.8 ± 18.4	<0.001
Diastolic BP (mmHg)	75.9 ± 11.0	75.7 ± 11.5	77.7 ± 11.2	77.2 ± 11.4	0.018
Fasting glucose (mg/dL)	99.9 ± 26.2	101.9 ± 29.5	100.7 ± 26.2	104.8 ± 38.2	0.149
Total cholesterol (mg/dL)	194.2 ± 31.6	195.7 ± 35.6	194.8 ± 36.9	194.2 ± 37.6	0.520
Triglyceride (mg/dL)	144.0 ± 109.2	154.8 ± 160.4	152.7 ± 137.1	136.7 ± 113.9	0.141
HDL-C(mg/dL)	52.1 ± 11.7	51.0 ± 12.3	51.1 ± 12.1	52.1 ± 14.2	0.298
AST (U/L)	28.3 ± 14.8	26.8 ± 14.4	25.9 ± 11.6	25.7 ± 14.4	<0.001
ALT (U/L)	36.1 ± 27.6	34.1 ± 29.9	30.7 ± 20.9	29.7 ± 22.2	<0.001
Creatinine (mg/dL)	1.15 ± 0.16	1.15 ± 0.16	1.18 ± 0.28	1.19 ± 0.28	0.003
eGFR (mL/min/1.73 m^2^)	77.1 ± 14.3	75.7 ± 14.0	73.8 ± 14.5	71.0 ± 14.9	<0.001
eGFR <60 mL/min/1.73 m^2^	42 (9.4)	51 (11.5)	68 (15.4)	101 (22.7)	<0.0001
Uric acid (mg/dL)	7.0 ± 1.37	7.1 ± 1.38	7.1 ± 1.78	6.8 ± 1.51	0.109
Total bilirubin (mg/dL)	0.86 ± 0.35	0.86 ± 0.38	0.86 ± 0.39	0.89 ± 0.43	0.955
Hemoglobin (g/dL)	15.1 ± 1.07	15.1 ± 1.23	15.0 ± 1.24	14.8 ± 1.37	<0.001
Previous CVD	6 (2.5)	9 (2.0)	11 (2.5)	20 (4.5)	0.0033
Hypertension	110 (24.7)	120 (27.0)	152 (34.3)	178 (40.0)	<0.0001
Diabetes mellitus	41 (9.2)	52 (11.7)	43 (9.7)	51 (11.5)	0.4660
Dyslipidemia	257 (57.8)	275 (61.9)	278 (62.8)	266 (59.8)	0.5069
Current smoker	145 (32.6)	139 (31.3)	162 (36.6)	138 (31.0)	0.9581
Habitual drinker	161 (36.2)	134 (30.2)	141 (31.8)	116 (26.1)	0.0034
**(B) Women**					
	**NLR Q1** **0.40–1.24** **(*n* = 267)**	**NLR Q2** **1.25–1.57** **(*n* = 267)**	**NLR Q3** **1.58–2.04** **(*n* = 268)**	**NLR Q4** **2.05–14.45** **(*n* = 267)**	***p* for Trend**
BMI (kg/m^2^)	23.5 ± 3.00	23.5 ± 3.19	23.7 ± 3.20	23.4 ± 3.13	0.679
Age (years)	48.4 ± 11.2	48.5 ± 11.6	49.5 ± 11.2	48.2 ± 11.3	0.604
Systolic BP (mmHg)	117.0 ± 18.1	118.3 ± 19.9	118.4 ± 19.8	118.8 ± 19.7	0.331
Diastolic BP (mmHg)	72.5 ± 11.2	73.8 ± 11.6	73.1 ± 12.0	72.7 ± 12.3	0.915
Fasting glucose (mg/dL)	95.1 ± 19.3	95.4 ± 22.3	95.9 ± 23.6	95.1 ± 22.5	0.973
Total cholesterol (mg/dL)	194.4 ± 37.0	195.0 ± 34.2	194.1 ± 40.0	187.8 ± 34.1	0.017
Triglyceride (mg/dL)	111.4 ± 99.8	100.6 ± 59.5	124.3 ± 323.4	99.3 ± 60.2	0.465
HDL-C (mg/dL)	64.1 ± 16.4	63.9 ± 15.7	63.1 ± 15.4	61.0 ± 14.6	0.104
AST (U/L)	25.1 ± 29.7	23.0 ± 11.5	25.1 ± 38.0	23.3 ± 15.9	0.088
ALT (U/L)	25.5 ± 40.1	23.4 ± 20.5	25.3 ± 53.4	23.2 ± 21.2	0.498
Creatinine (mg/dL)	0.86 ± 0.13	0.88 ± 0.15	0.90 ± 0.44	0.95 ± 0.87	0.460
eGFR (mL/min/1.73 m^2^)	81.9 ± 15.3	80.4 ± 15.8	80.2 ± 16.8	81.7 ± 18.2	0.560
eGFR <60 mL/min/1.73 m^2^	17 (6.4)	19 (7.1)	28 (10.5)	22 (8.2)	0.2291
Uric acid (mg/dL)	5.4 ± 1.25	5.5 ± 1.29	5.4 ± 1.27	5.5 ± 1.36	0.821
Total bilirubin (mg/dL)	0.70 ± 0.32	0.65 ± 0.29	0.65 ± 0.25	0.68 ± 0.30	0.856
Hemoglobin (g/dL)	12.8 ± 1.40	13.0 ± 1.06	13.0 ± 1.21	12.9 ± 1.47	0.246
Previous CVD	2 (0.8)	4 (1.5)	6 (2.2)	4 (1.5)	0.3684
Hypertension	62 (23.2)	68 (25.5)	63 (23.5)	64 (24.0)	0.9806
Diabetes mellitus	14 (5.2)	14 (5.2)	18 (6.7)	17 (6.4)	0.4523
Dyslipidemia	153 (57.3)	151 (56.6)	149 (55.6)	128 (47.9)	0.0332
Current smoker	17 (6.4)	12 (4.5)	7 (2.6)	6 (2.3)	0.0074
Habitual drinker	15 (5.6)	15 (5.6)	10 (3.7)	11 (4.1)	0.2740

Values are expressed as the mean ± standard deviation, median (interquartile range), and number (percentage). BMI, body mass index; BP, blood pressure; HDL-C, high-density lipoprotein cholesterol. AST, aspartate aminotransferase; ALT, alanine aminotransferase; CVD, cardiovascular disease (including stroke and myocardial infarction).

**Table 3:** NLR quartile percentages in normal-weight and overweight/obese men and women with and without CKD.

	Normal Weight			Overweight/Obese		
	Non-CKD *n* (%)	CKD *n* (%)	*p*-Value	Non-CKD *n* (%)	CKD *n* (%)	*p*-Value
NLR quartiles (men)			0.020			<0.001
0.40–1.28	155 (23.7%)	17 (15.3%)		248 (28.8%)	25 (16.6%)	
1.29–1.62	157 (24.0%)	19 (17.1%)		236 (27.4%)	32 (21.2%)	
1.63–2.05	161 (24.6%)	31 (27.9%)		214 (24.9%)	37 (24.5%)	
2.06–8.70	182 (27.8%)	44 (39.6%)		162 (18.8%)	57 (37.8%)	
NLR quartiles (women)			0.995			0.122
0.40–1.24	147 (23.7%)	9 (22.5%)		103 (28.5%)	8 (17.4%)	
1.25–1.57	162 (26.1%)	11 (27.5%)		86 (23.8%)	8 (17.4%)	
1.58–2.04	151 (24.3%)	10 (25.0%)		89 (24.6%)	18 (39.1%)	
2.05–14.45	161 (25.9%)	10 (25.0%)		84 (23.2%)	13 (26.1%)	

**Table 4:** Odds Ratios for CKD Stratified by BMI Status in All participants (**A**), Men (**B**), and Women (**C**).

(A) All Participants				
	18.5 ≤ BMI < 24 (*n* = 1427)		BMI ≥ 24 (*n* = 1419)	
Variable	OR (95% CI)	*p*-Value	OR (95% CI)	*p*-Value
Age (year)	1.10 (1.08, 1.13)	<0.001	1.15 (1.12, 1.17)	<0.001
BMI (kg/m^2^)	1.10 (0.95, 1.29)	0.198	1.03 (0.95, 1.12)	0.407
Sex (men vs. women)	2.62 (1.43, 4.82)	0.002	1.17 (0.67, 2.02)	0.580
Current smoker	0.97 (0.54, 1.71)	0.904	1.52 (0.92, 2.49)	0.099
Habitual drinker	0.56 (0.30, 1.06)	0.076	0.75 (0.46, 1.22)	0.247
Systolic blood pressure (mmHg)	1.008 (0.997, 1.020)	0.155	1.006 (0.996, 1.016)	0.213
Fasting plasma glucose (mg/dL)	0.989 (0.978, 1.001)	0.063	0.991 (0.983, 1.000)	0.040
Total cholesterol (mg/dL)	1.009 (1.003, 1.015)	0.003	0.999 (0.993, 1.006)	0.859
Triglyceride (mg/dL)	1.000 (0.996, 1.003)	0.879	0.999 (0.997, 1.001)	0.198
HDL-C (mg/dL)	1.008 (0.992, 1.024)	0.310	0.978 (0.960, 0.997)	0.024
ALT (U/L)	0.999 (0.992, 1.007)	0.863	1.005 (0.999, 1.011)	0.090
Uric acid (mg/dL)	1.54 (1.32, 1.78)	<0.001	1.59 (1.40, 1.81)	<0.001
Total bilirubin (mg/dL)	0.46 (0.25, 0.83)	0.010	0.55 (0.32, 0.95)	0.031
Hemoglobin (g/dL)	0.70 (0.60, 0.81)	<0.001	0.95 (0.81, 1.11)	0.500
Neutrophil/lymphocyte ratio (NLR)	0.96 (0.77, 1.20)	0.744	1.45 (1.13, 1.84)	0.003
**(B) Men**				
	**18.5 ≤ BMI < 24 (*n* = 766)**		**BMI ≥ 24 (*n* = 1011)**	
**Variable**	**OR (95% CI)**	***p*** **-Value**	**OR (95% CI)**	***p*** **-Value**
Age (year)	1.10 (1.07, 1.13)	<0.001	1.15 (1.12, 1.18)	<0.001
BMI (kg/m^2^)	1.18 (0.98, 1.42)	0.080	1.02 (0.92, 1.13)	0.692
Current smoker	0.95 (0.53, 1.72)	0.874	1.55 (0.92, 2.60)	0.097
Habitual drinker	0.60 (0.31, 1.16)	0.130	0.78 (0.47, 1.29)	0.328
Systolic blood pressure (mmHg)	1.007 (0.993, 1.021)	0.332	1.004 (0.992, 1.016)	0.500
Fasting plasma glucose (mg/dL)	0.992 (0.980, 1.004)	0.187	0.988 (0.978, 0.999)	0.026
Total cholesterol (mg/dL)	1.012 (1.004, 1.020)	0.002	1.002 (0.995, 1.009)	0.599
Triglyceride (mg/dL)	1.000 (0.996, 1.004)	0.863	0.999 (0.996, 1.001)	0.213
HDL-C (mg/dL)	1.002 (0.982, 1.022)	0.861	0.966 (0.943, 0.989)	0.004
ALT (U/L)	0.978 (0.959, 0.998)	0.028	1.002 (0.992, 1.011)	0.728
Uric acid (mg/dL)	1.47 (1.23, 1.77)	<0.001	1.56 (1.35, 1.80)	<0.001
Total bilirubin (mg/dL)	0.54 (0.28, 1.04)	0.064	0.65 (0.36, 1.17)	0.153
Hemoglobin (g/dL)	0.66 (0.55, 0.80)	<0.001	0.92 (0.76, 1.10)	0.355
Neutrophil/lymphocyte ratio (NLR)	0.97 (0.74, 1.29)	0.846	1.37 (1.03, 1.82)	0.030
**(C) Women**				
	**18.5 ≤ BMI < 24 (*n* = 661)**		**BMI ≥ 24 (*n* = 408)**	
**Variable**	**OR (95% CI)**	***p*** **-Value**	**OR (95% CI)**	***p*** **-Value**
Age (year)	1.12 (1.07, 1.17)	<0.001	1.15 (1.10, 1.21)	<0.001
BMI (kg/m^2^)	0.95 (0.72, 1.25), 0.720	0.720	1.05 (0.92, 1.20)	0.469
Current smoker	- ^†^	-	0.64 (0.04, 9.26)	0.741
Habitual drinker	- ^†^	-	0.50 (0.04, 6.14)	0.589
Systolic blood pressure (mmHg)	1.010 (0.990, 1.032)	0.330	1.013 (0.994, 1.032)	0.173
Fasting plasma glucose (mg/dL)	0.985 (0.953, 1.019)	0.396	1.002 (0.988, 1.016)	0.825
Total cholesterol (mg/dL)	1.004 (0.993, 1.014	0.510	0.990 (0.976, 1.003)	0.142
Triglyceride (mg/dL)	1.001 (0.994, 1.008)	0.849	1.000 (0.995, 1.005)	0.933
HDL-C (mg/dL)	1.019 (0.991, 1.048)	0.186	1.012 (0.976, 1.049)	0.529
ALT (U/L)	1.003 (0.998, 1.008)	0.207	1.005 (0.997, 1.013)	0.204
Uric acid (mg/dL)	1.77 (1.34, 2.35)	<0.001	1.71 (1.27, 2.31)	<0.001
Total bilirubin (mg/dL)	0.31 (0.07, 1.29)	0.108	0.21 (0.04, 1.25)	0.087
Hemoglobin (g/dL)	0.76 (0.58, 0.98)	0.032	1.08 (0.78, 1.49)	0.641
Neutrophil/lymphocyte ratio (NLR)	0.87 (0.53, 1.45)	0.601	1.77 (1.08, 2.90)	0.023

Multivariable logistic regression analysis after adjustment of confounders; ^†^ there was no CKD in current smokers and habitual drinkers.

**Table 5:** Regression Analysis of eGFR (mL/min/1.73 m^2^) against Neutrophil/Lymphocyte Ratio (NLR) Stratified by BMI Status in Men (**A**) and Women (**B**).

(A) Men				
	18.5 ≤ BMI < 24 (*n* = 766)		BMI ≥ 24 (*n* = 1011)	
Variable	β (95% CI)	*p*-Value	β (95% CI)	*p*-Value
Age (year)	−0.62 (−0.70, −0.54)	<0.001	−0.70 (−0.78, −0.63)	<0.001
BMI (kg/m^2^)	−0.62 (−1.25, 0.01)	0.054	−0.067 (−0.408, 0.275)	0.700
Current smoker	0.67 (−1.28, 2.62)	0.501	−0.85 (−2.54, 0.84)	0.325
Habitual drinker	1.81 (−0.20, 3.83)	0.078	2.00 (0.34, 3.66)	0.018
Systolic blood pressure (mmHg)	−0.008 (−0.062, 0.046)	0.782	−0.042 (−0.086, 0.003)	0.066
Fasting plasma glucose (mg/dL)	0.026 (−0.001, 0.052)	0.059	0.039 (0.012, 0.066)	0.005
Total cholesterol (mg/dL)	−0.048 (−0.075, −0.021)	0.001	−0.006 (−0.030, 0.018)	0.628
Triglyceride (mg/dL)	0.008 (−0.004, 0.020)	0.178	0.007 (0.002, 0.013)	0.011
HDL-C (mg/dL)	−0.007 (−0.082, 0.068)	0.854	0.080 (0.006, 0.154)	0.035
ALT (U/L)	0.046 (0.009, 0.083)	0.016	0.031 (0.002, 0.060)	0.038
Uric acid (mg/dL)	−1.60 (−2.18, −1.02)	<0.001	−2.14 (−2.65, −1.63)	<0.001
Total bilirubin (mg/dL)	2.81 (0.59, 5.03)	0.013	−0.89 (−2.83, 1.06)	0.372
Hemoglobin (g/dL)	0.39 (−0.34, 1.11)	0.298	0.11 (−0.55, 0.77)	<0.001
Neutrophil/lymphocyte ratio (NLR)	−0.26 (−1.37, 0.86)	0.652	−0.41 (−1.51, 0.69)	0.464
**(B) Women**				
	**18.5 ≤ BMI < 24 (*n* = 661)**		**BMI ≥ 24 (*n* = 408)**	
**Variable**	**β (95%** **CI)**	***p*** **-Value**	**β (95%** **CI)**	***p*** **-Value**
Age (year)	−0.72 (−0.84, −0.61)	<0.001	−0.89 (−1.04, −.074)	<0.001
BMI (kg/m^2^)	0.20 (−0.56, 0.97)	0.602	0.27 (−0.29, 0.83)	0.341
Current smoker	−5.45 (−10.50, −0.41)	0.034	1.96 (−6.62, 10.55)	0.653
Habitual drinker	0.88 (−4.14, 5.89)	0.731	−0.34 (−0.06, 0.08)	0.916
Systolic blood pressure (mmHg)	−0.025 (−0.094, 0.043)	0.466	0.009 (−0.063, 0.081)	0.806
Fasting plasma glucose (mg/dL)	0.068 (−0.015, 0.150)	0.109	0.016 (−0.034, 0.066)	0.526
Total cholesterol (mg/dL)	−0.004 (−0.041, 0.032)	0.813	0.039 (−0.008, 0.086)	0.103
Triglyceride (mg/dL)	−0.008 (−0.031, 0.016)	0.526	−0.005 (−0.005, 0.008)	0.653
HDL-C (mg/dL)	−0.117 (−0.198, −0.037)	0.004	0.041 (−0.069, 0.151)	0.463
ALT (U/L)	0.002 (−0.024, 0.028)	0.856	−0.012 (−0.057, 0.032	0.588
Uric acid (mg/dL)	−3.46 (−4.40, −2.52)	<0.001	−2.93 (−4.00, −1.86)	<0.001
Total bilirubin (mg/dL)	1.53 (−2.02, 5.08)	0.396	8.04 (2.82, 13.27)	0.003
Hemoglobin (g/dL)	1.06 (0.26, 1.87)	0.009	0.48 (−0.65, 1.60)	0.408
Neutrophil/lymphocyte ratio (NLR)	0.075 (−1.04, 1.20)	0.895	−0.73 (−2.69, 1.22)	0.461

Multivariable linear regression analysis after adjustment of confounders; β indicates the change in eGFR (mL/min/1.73 m^2^) per unit of covariate increase.

## Data Availability

Not applicable.
